# Substrate availability and toxicity shape the structure of microbial communities engaged in metabolic division of labor

**DOI:** 10.1002/mlf2.12025

**Published:** 2022-06-30

**Authors:** Miaoxiao Wang, Xiaoli Chen, Yue‐Qin Tang, Yong Nie, Xiao‐Lei Wu

**Affiliations:** ^1^ Department of Energy & Resources Engineering, College of Engineering Peking University Beijing China; ^2^ Department of Environmental Systems Science ETH Zürich Zürich Switzerland; ^3^ Department of Environmental Microbiology Eawag Dübendorf Switzerland; ^4^ Department of Environmental Science and Engineering, College of Architecture and Environment Sichuan University Chengdu China; ^5^ Institute of Ocean Research Peking University Beijing China; ^6^ Institute of Ecology Peking University Beijing China

**Keywords:** community structure, mathematical model, metabolic division of labor, substrate, synthetic microbial consortium

## Abstract

Metabolic division of labor (MDOL) represents a widespread natural phenomenon, whereby a complex metabolic pathway is shared between different strains within a community in a mutually beneficial manner. However, little is known about how the composition of such a microbial community is regulated. We hypothesized that when degradation of an organic compound is carried out via MDOL, the concentration and toxicity of the substrate modulate the benefit allocation between the two microbial populations, thus affecting the structure of this community. We tested this hypothesis by combining modeling with experiments using a synthetic consortium. Our modeling analysis suggests that the proportion of the population executing the first metabolic step can be simply estimated by Monod‐like formulas governed by substrate concentration and toxicity. Our model and the proposed formula were able to quantitatively predict the structure of our synthetic consortium. Further analysis demonstrates that our rule is also applicable in estimating community structures in spatially structured environments. Together, our work clearly demonstrates that the structure of MDOL communities can be quantitatively predicted using available information on environmental factors, thus providing novel insights into how to manage artificial microbial systems for the wide application of the bioindustry.

## 1 INTRODUCTION

In natural environments, microorganisms rarely live autonomously; instead, they interact with other individuals to form complex communities, in which they secrete a variety of toxins to compete with each other, or share metabolites to mutually benefit their survival. Among diverse modes of microbial interaction, metabolic division of labor (MDOL) is one of the most widespread phenomena, where distinct populations perform different but complementary steps of the same metabolic pathway^1^, ^4^. MDOL controls numerous ecologically and environmentally important biochemical processes. One important aspect of microbial metabolism implemented by MDOL is the degradation of a variety of complex organic compounds. Bacterial degradation of these complex substrates is usually mediated by long metabolic pathways via a number of intermediates. Earlier studies based on multiomics suggested that a number of these degradation pathways are segregated across different members within a natural community in an MDOL manner. Typical examples include cooperative lignocellulose breakdown in goat gut microbiomes^5^, plant polysaccharide digestion through MDOL in honey bee gut microbiota^6^, as well as the degradation of polycyclic aromatic hydrocarbons (PAHs) via sequential cross‐feeding between marine microorganisms^7^, ^8^. Owing to these important ecological functions contributed by MDOL, it is critical to understand how the communities engaged in MDOL are regulated in detail.

However, omics‐based studies have so far failed to provide a direct solution due to the complex set of factors involved in a natural community. Recent progress in synthetic ecology offers a “bottom‐up” approach to investigate the ecological dynamics of MDOL in simple microbial systems^4^, ^9^, ^10^. Although many studies have constructed synthetic consortia to engage in MDOL for the removal of organic pollutants^11^, ^16^, they usually focused on whether MDOL enhances the biodegradation efficiency compared to relevant monocultures comprised of single species. Another challenge in understanding the dynamics of these systems is that suitable quantitative models explaining these systems remain absent. Therefore, a quantitative framework to forecast how important ecological factors regulate the structure of a community engaged in MDOL is urgently required^17^.

The substrate whose concentration spatially and temporally fluctuates in the marine^18^, soil^19^, and wastewater^20^ environments acts as one of the most important conditions that govern the performance of the microbial communities^21^, ^23^. First, the concentration of substrates regulates the growth of microbial populations according to the Monod equation^24^. Second, many substrates, such as PAHs^25^, ^26^, pesticides^11^, ^14^, and antibiotics^16^, are toxic to bacterial cells, inhibiting their growth. Increasing the substrate concentration enhances resource availability of a population, which not only benefits its growth but also potentially increases the toxic effects of substrate that harms its growth (e.g., growth kinetics may follow the equations integrated with toxicity terms^27^). Thus, the concentration and toxicity of substrate profoundly affect the fitness of its microbial degraders^26^, ^28^, ^29^. However, how substrate concentration and toxicity affect the relative fitness of different strains involved in a community and thus govern the structure of the community, still remains ill‐defined. As the structure of a community is fundamental to determining its functioning^30^, ^31^, solving this question is fundamental for managing such microbial systems for the removal of serious pollutants.

**Figure 1:**
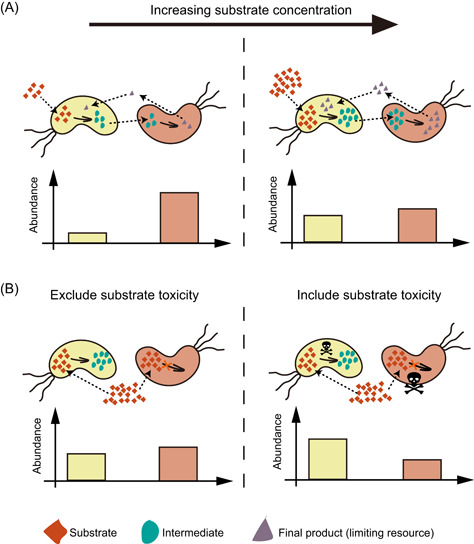
Hypothesis for how substrate concentration and toxicity govern the structure of the community engaged in metabolic division of labor (MDOL). In a community degrading an organic compound through MDOL, the final product was assumed to be the sole carbon source and was synthesized by the strain performing the second step. Therefore, this strain will obtain more nutrients, while the other strain has to collect products released from this population. Thus, the last population was named “Embezzler” (denoted as the red cells). (A) Increasing the concentration of the substrate improves the flux of the pathway. Since the product consumption ability of Embezzler cells is limited, increasing the concentration will lead to higher final product leakiness, favoring the growth of the first population. (B) Introducing substrate biotoxicity also favors the first population because it converts this toxic substrate (denoted as skull and bones), resulting in lower intracellular substrate concentration compared to that of the Embezzler cells. Thus, the first population was named “Detoxifier.”

Distinct from the pure culture, the effects of substrate on different populations involved in an MDOL community may vary quite a lot. First, asymmetric benefit allocation exists between different populations in the MDOL community. In MDOL communities that degrade organic compounds, only the population performing the last steps can produce the growth resources (such as small organic acids) that support the bacterial growth (several examples are given in Figure S1). Therefore, the population performing the last steps can preferentially acquire and privatize these nutrients (which we henceforth call *product privatization*), thus acquiring the most benefit, while the other members have to collect nutrients leaked from this population (Figure 1A). This uneven allocation of limited resources generally benefits the population that executes the last steps (we henceforth name this population the “Embezzler”, analogous to a human worker responsible for the final step of an assembly line, who pockets the final product and fails to share profits with other workers). This phenomenon has been observed in many recent studies^11^, ^14^, ^32^. Increasing substrate concentration would enhance the flux of metabolites^33^, ^34^. As the Embezzler only has a limited capacity for consuming the final product, increased metabolic flux causes more product released from the Embezzler cells, in turn facilitating the growth of the other population (Figure 1A; the right panel). Second, substrate toxicity exerts different effects on the individual members of the MDOL community. The population performing the first step transforms the toxic substrate to intermediates (named here “Detoxifier” henceforth), thus reducing its intracellular concentration of the toxic substrate (Figure 1B). Transformation of the toxic substrate considerably reduces the effects of the toxic substrate on the Detoxifier rather than on the Embezzler. As a result, the Detoxifier population is favored whenever the substrate is toxic to both Detoxifier and Embezzler.

It is critical to better understand the effects of substrate concentration and toxicity on the structure of an MDOL community. To test our two hypotheses and to assess how substrate concentration and toxicity shape the structure of microbial community engaged in MDOL, we combined mathematical modeling and experimentation using a synthetic microbial community. We also tested whether the effects of substrate concentration and toxicity change when such a community grows in spatially well‐mixed and structured environments.

## 2 RESULTS

### 2.1 Substrate availability and toxicity shape the structure of the MDOL communities in a well‐mixed system

#### 2.1.1 An ordinary differential equation (ODE) model for modeling the dynamics of a community engaged in MDOL

**Figure 2:**
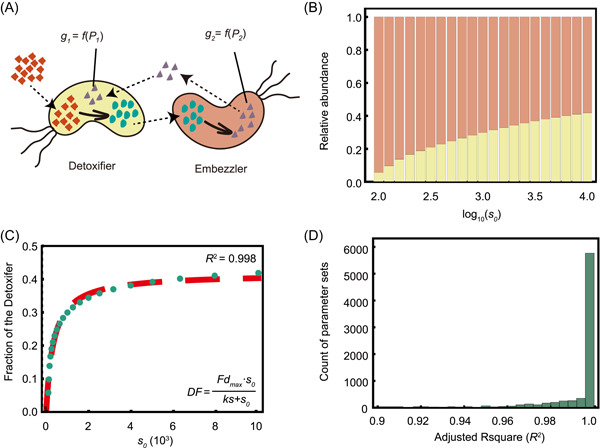
Simulation of the ordinary differential equation (ODE) model excluding substrate toxicity. (A) Schematic diagram showing the basic assumptions of our ODE model omitting substrate toxicity. (B, C) A representative case shows how substrate concentration affects the structure of a metabolic division of labor (MDOL) community. (B) The steady‐state structure of the simulated MDOL community with the conditions of different initial substrate concentrations. As denoted in (A), the yellow bars indicate the relative abundance of Detoxifier, while the red bars indicate the relative abundance of Embezzler. (C) The relationship between substrate concentration and the steady‐state fraction of Detoxifier. The green dotted line denotes the simulated steady‐statefraction of the Detoxifier, while the red dashed line shows the plot of the best fitting function using Equation (1). Parameter values used in these simulations are as follows: *y* = 10^−4^, *Cp* = 10, *bg* = 1, *α*_1_ = 10,000, *α*_2_ = 1000, *β*_2_ = 1, $\gamma_{s}$ = 1, $\gamma_{i}$ = 1, $\gamma_{p}$ = 1, *ρ = *10^−2^. The best‐fitting value of *ks*, in this case, is 35.3, and that of *Fd*_*max*_ is 0.417. (D) Distributions of adjusted *R* ^2^ of the fitting functions in the second‐round simulations that include substrate toxicity, using 7776 parameter value combinations of the five key parameters ($\alpha_{1}$, $\gamma_{s}$, $\gamma_{i}$, $\gamma_{p}$, and Cp).

To assess the effects of substrate concentration and its toxicity on the structure of the MDOL communities, we simulated the dynamics of a community engaged in MDOL in a well‐mixed system using a mathematical model. The dimensionless form of this model is composed of 11 ODEs (Equations (4), (5), (6), (7), (8), (9), (10), (11), (12), (13) in Materials and Methods section; Table S1 and S2). As summarized in Figure 2A, we considered the degradation of an organic substrate (*S*) into an intermediate metabolite (*I*), before being degraded to the final product (*P*). We assumed that two strains carry out this pathway via MDOL, with the first strain only executing the first step, and the second only executing the second. Initially, only *S* was supplied and the initial concentration was parameterized by $s_{0}$ (nondimensional). Importantly, based on our hypothesis of “Embezzler behavior,” we assumed that *P*, which is synthesized by the second strain, is the solely available resource for the growth of both strains. As a result, the second strain obtained the advantage of preferentially acquiring the resource, while the first strain only obtained the growth‐limiting resource that was leaked from the second strain. Therefore, the second strain in our model system behaved like an “Embezzler”. Moreover, to assess the effects of substrate toxicity, we imposed a term in the equation describing population growth^27^ (Table S3) to the growth function, and the degree of toxicity was mediated by parameter *θ*. Thus, for the scenarios where the substrate was assumed to be toxic, the strain executing the first step behaved like a “Detoxifier”. Details about the model are described in Supporting Information: S1.1–S1.4.

#### 2.1.2 Analysis of the ODE model indicates that initial substrate concentration affects the structure of an MDOL community

To test our first hypothesis, which states that substrate concentration affects the structure of the community, we analyzed our ODE model, omitting substrate toxicity (Figure 2A). As the dimensionless model contains 11 independent parameters (Table S4) potentially affecting the structure of an MDOL community, we performed the first round of numerical simulations using 885,735 parameter sets considering realistic value ranges for all parameters (Supporting Information: S1.3; Table S4). Our analysis showed that the Embezzler population dominated the steady‐state community in these simulations, that is, steady‐state frequencies of Detoxifier are lower than 0.5 (Figures 2B and S2; no toxic scenarios), which was in agreement with our basic assumption of product privatization. Next, we performed multivariate regression analyses, which confirmed that six key parameters played vital roles in shaping the structure of the MDOL community (Table S4 and Figure S3A; *p* < 0.01 and the fitting coefficient values over 0.01). Notably, $s_{0}$ was the second most important according to the absolute value of the fitting coefficient. $s_{0}$ positively correlated with the steady‐state proportion of the Detoxifier population, suggesting that a higher initial substrate concentration favors the Detoxifier. These observations are consistent with our first hypothesis.

Following the second round of simulations (Supporting Information: S1.3), we found that when all other five key parameters were kept constant, the steady‐state proportion of the Detoxifier population (DF) increased with an increase in the initial substrate concentration (Figure 2B,C). This steady‐state proportion can be estimated using a Monod‐like formula with $s_{0}$ as the function argument (Figure 2C),

(1) $$\text{DF}=\frac{{\text{Fd}}_{\max}\text{∙}s_{0}}{\text{ks}\text{+}s_{0}}$$

Here, ${\mathrm{Fd}}_{\max}$ represents the maximum proportion of the Detoxifier populations when the substrate is nontoxic; ks represents the half‐saturation constant. Our analysis indicated that the simulation results of all tested parameter sets can be accurately fit to Equation (1) (Figure 2D; values of adjusted *R* ^2^ mostly over 0.95), although the best‐fit values of ${\mathrm{Fd}}_{\max}$ and ks were affected by the values of the other five key parameters (Supporting Information: S1.3; Table S5; Figures S4 and S5). Together, these results suggest that, in the absence of substrate toxicity, the proportion of the Detoxifier population increases nonlinearly with the increase of the initial substrate concentration and maintains a maximum value.

To investigate how substrate concentration governs the structure of a community, we next analyzed the intracellular and extracellular concentrations of the final product of the two populations. We found that the fraction of final product released by the Embezzler population increased with the increase of initial substrate concentration (Figure S6A–I, red dots). As a consequence, the Detoxifier obtained more product from the environment, resulting in a higher intracellular product concentration, gradually approaching that of the Embezzler. Moreover, based on the first hypothesis, the intracellular product concentration of the Detoxifier should never exceed that of the Embezzler, even if we raised the substrate concentration to high levels. This prediction was confirmed by our analyses (Figure S6A–I, blue dots). As a consequence, Embezzler cells maintained their advantage by privatizing the final product. This result suggests that in the absence of substrate toxicity, the benefit from product privatization obtained by the Embezzler population cannot be completely eliminated by simply increasing the substrate concentration. This observation matched with our result that the maximum proportion of the Detoxifier population (${\mathrm{Fd}}_{\max}$) never exceeded 0.5 (Figure S5). In summary, these results suggest that substrate concentration affects the structure of the community engaged in MDOL by affecting the amount of the final product released by the Embezzler (Figure 1A).

#### 2.1.3 Analysis of the ODE model indicates that substrate toxicity affects the structure of an MDOL community

**Figure 3:**
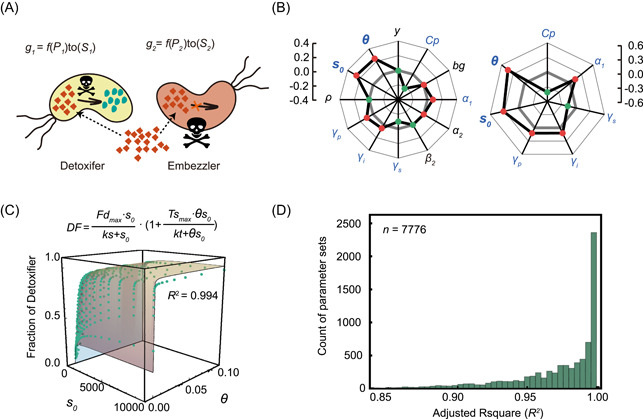
Simulation using the ordinary differential equation (ODE) model suggests that both substrate concentration and its toxicity affect the structure of a community engaged in metabolic division of labor (MDOL). (A) Schematic diagram showing the basic assumptions of our ODE model that includes substrate toxicity. (B) Multiple linear regression analysis of the simulation results of the ODE model showed how the parameters included in the model affect the structure of the MDOL community. Left: results from the first‐round simulations that considered all the 12 parameters are shown. Blue font denotes the identified key parameters. Right: results from the second‐round simulations that only considered the seven‐key parameters. The axis of the radar plot denotes the values of fitting coefficients of the parameters from multiple linear regression analyses. Red dots denote that the corresponding parameter is positively correlated with the steady‐state fraction of the Detoxifier, while the green dots represent the negative correlation. The origin axis (0) is highlighted by gray bold lines to emphasize the fact that the closer a value is to zero, the smaller the effect on the community structure by the corresponding parameter. The data are also listed in Tables S4 and S5. In this analysis, the toxic effects of substrate on population growth were assumed to follow a reciprocal relationship. Results considering other relationships are shown in Figure S3. (C) A representative case shows how both substrate concentration and its toxicity collectively affect the steady‐state proportion of Detoxifier cells. The green dots denote the simulated steady‐state fraction of the Detoxifier, and the surface shows the plot of the best fitting function using Equation (2). Parameter values used in these simulations: *y* = 10^−4^, *Cp* = 10, *bg* = 1, *α*_1_ = 10,000, *α*_2_ = 1000, *β*_2_ = 1, $\gamma_{s}$ = 1, $\gamma_{i}$ = 1, $\gamma_{p}$ = 1, *ρ = *10^−2^. The best‐fitting value of *k*s, *Fd*_*max*_, *kt*, and *TS*_*max*_, in this case, are 48.9, 0.423, 0.848, and 3.39, respectively. (D) Distributions of adjusted *R* ^2^ of the fitting functions in the second‐round simulations that include substrate toxicity, using 7776 parameter value combinations of the five key parameters ($\alpha_{1}$, $\gamma_{s}$, $\gamma_{i}$, $\gamma_{p}$, and Cp).

To test our second hypothesis, we next employed an ODE model that included the parameter of substrate toxicity (Figure 3A). We applied a similar simulation and analysis method to that used in the previous section (Supporting Information: S1.3), we found that the degree of toxicity (*θ*) of the substrate also played a significant role in structuring the MDOL community. *θ* exhibited a significantly positive relationship with the final proportion of the Detoxifier population (Figures 3B, S2 and Table S4), in agreement with our second hypothesis. We then enhanced Equation (1) to collectively consider the effects of substrate concentration and its toxicity (Figure 3C), as follows:

(2) $$\text{DF}=\frac{{\text{Fd}}_{\max}\text{∙}s_{0}}{\text{ks}\text{+}s_{0}}\text{∙}\left(1\text{+}\frac{{\text{Ts}}_{\max}\text{∙}{\text{θs}}_{0}}{\text{kt}\text{+}{\text{θs}}_{0}}\right)$$

In Equation (2), we use the term $1+\frac{{\mathrm{Ts}}_{\max}∙\theta s_{0}}{\mathrm{kt}+\theta s_{0}}$ to describe the effect of substrate toxicity on the proportion of the Detoxifier populations. ${\mathrm{Ts}}_{\max}$ represents the maximum fold increase of Detoxifier proportion due to substrate toxicity; ks represents the half‐saturation constant of substratetoxicity. This term is positively affected by the degree of toxicity (*θ*) and the substrate concentration ($s_{0}$) because increasing either the degree off toxicity or substrate concentration inhibited population growth (see Equations (12 and 13) in Materials and Methods section and Table S3). Our analyses further indicated that the Detoxifier population values derived from numerical simulations accurately match to the values predicted by Equation (2) (Figure 3D; values of adjusted *R* ^2^ mostly over 0.90; see Table S5 and Figures S7–S10 for parameter sensitive analyses). These results suggest that when substrate toxicity is taken into account, the proportion of the Detoxifier population increases with both the initial concentration and the degree of toxicity of the substrate.

To address why substrate toxicity affects the structure of our model community, we next analyzed the intracellular and extracellular concentrations of both *S* and *P* of the two populations. As shown in Figure S11, the fraction of final product released by the Embezzler population largely agrees with the results derived from nontoxic scenarios, suggesting that the presence of substrate toxicity fails to increase the leakiness of the final product from the Embezzler. Our analysis of the *S* concentration showed that the Detoxifier population generally maintained a lower intracellular concentration level of *S* than that of the Embezzler (Figure S12), due to its conversion of *S*, thus possessing a growth advantage over the Embezzler population. Based on this mechanism, a higher rate of the first reaction, or lower *S* transport rate, appears to favor the Detoxifier population because these two conditions assist Detoxifier in maintaining a lower intracellular *S* concentration. Consistent with this corollary, we found that ${\mathrm{Ts}}_{\max}$ was significantly positively correlated with $a_{1}$ and significantly negatively correlated with $\gamma_{s}\;$(Table S5 and Figure S10). Together, these results indicated that the difference in intracellular concentration of substrate is the main reason why substrate toxicity favors the Detoxifier population (Figure 1B).

When we assessed the community structure under different conditions, we found that the Detoxifier population dominated the community when the substrate concentration and substrate toxicity were sufficiently high (relative proportion of the Detoxifier exceeded 50% of the community; Figures 3C and S2), suggesting that the benefit from product privatization of the Embezzler can be neutralized by higher substrate concentrations and toxicity. This phenomenon is quantitatively characterized by Equation (2): the maximum Detoxifier proportion (${\mathrm{Fd}}_{\max}$) never exceeded 0.5 in the absence of substrate toxicity (Figure S8), but substrate toxicity can allow Detoxifier in breaking through this constraint, as quantified by the term $1+\frac{{\mathrm{Ts}}_{\max}∙\theta s_{0}}{\mathrm{kt}+\theta s_{0}}$.

In summary, our simulations clearly showed that when a compound degradation pathway is executed through MDOL in a community, both increasing substrate concentration and toxicity of the substrate favor the Detoxifier population, resulting in different community structures.

#### 2.1.4 Experimental evaluation of our model prediction using a liquid culture of a synthetic microbial consortium engaged in MDOL

**Figure 4:**
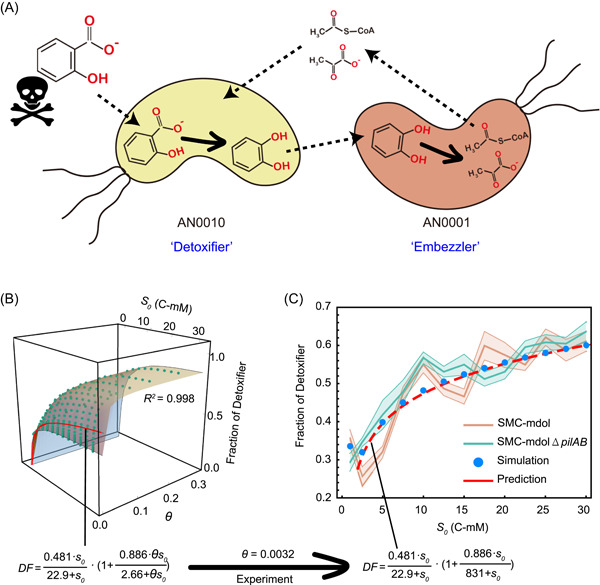
Structure of SMC‐mdol in a spatially unstructured system governed by different substrate concentrations and toxicity. (A) Schematic showing the metabolic division of labor between strain *Pseudomonas stutzeri* AN0010 and strain *P. stutzeri* AN0001 during salicylate degradation. Strain AN0010 degrades salicylate into the intermediate catechol, which feeds strain AN0001 as the substrate for further degradation. However, strain AN0010 cannot use a direct carbon source from salicylate degradation to support its growth. When AN0010 was paired with strain AN0001, AN0001 degrades catechol to pyruvate and acetyl‐CoA, enlabling the growth of AN0010. The skull and bones sign indicates that salicylate is toxic. (B) Predicting the structure of the synthetic consortium using our ordinary differential equation model, as well as the derived predictive function using Equation (2). The relationship between the steady‐state fraction of the Detoxifier population and substrate concentration (*s*_*0*_), as well as the degree of substrate toxicity (*θ*), was built from our mathematical model using parameters consistent with our experimental system. Each green dot shows the steady‐state fraction of the Detoxifier obtained by one simulation associated with the specific parameter set. The surface diagram shows the distribution of the steady‐state fraction of the Detoxifier predicted by our proposed simple formula. The red line on the surface denotes the scenario *θ *= 0.0032, which is the toxic strength of salicylate obtained from experimental measurements. (C) The experimental measured steady‐state fractions of Detoxifier in cultures with different salicylate concentrations are consistent with those from mathematical predictions. Experiments were performed in six replicates. The translucent band indicates the error bar of the data. Note that in the plots, substrate concentrations are shown in dimensional form (*S*_*0*_, C‐mM), but in the predictive functions, the fitting analysis was performed using its dimensionless form (*s*_*0*_).

To experimentally test the prediction from our ODE model, we engineered a synthetic consortium composed of two *Pseudomonas stutzeri* strains, which cooperatively degrade an organic compound, salicylate, via MDOL (Figure 4A). In this synthetic consortium, strain *P. stutzeri* AN0010 only retained its ability to convert the toxic substrate, salicylate, to the intermediate catechol^35^, behaving like the “Detoxifier”. The second strain, *P. stutzeri* AN0001, was only able to metabolize catechol but possessed the preferential access to the final product, that is, pyruvate and acetyl‐CoA (Figure 4A), the direct carbon source of both strains, thus behaving like the “Embezzler”. For simplicity, we henceforth refer to our community as “SMC‐mdol”.

We first derived a function to predict the structure of our synthetic consortium based on our model using experimentally measured or previously reported parameters (Figure 4B and Table S6; Supporting Information: S1.3). We quantified the toxicity of salicylate and the measured dimensionless value of the degree of toxicity (θ) of salicylate was 0.0032 (Figure S13). On the basis of this measurement, we mathematically predicted the effects of substrate concentration on the structure of SMC‐mdol, as indicated by the red line in Figure 4B,C. In the liquid minimal medium supplemented with different concentrations of salicylate, SMC‐mdol exhibited similar dynamics to that of our corresponding ODE simulations (Figure S14). The steady‐state proportion of Detoxifier population increased from 25.6 ± 2.5% to 61.1 ± 2.6% as a function of initial salicylate concentration (Figure 4C). Moreover, our prediction function accurately estimated the steady‐state structure of SMC‐mdol, with a predictive power (adjusted *R* ^2^) of 0.983. Importantly, when the substrate concentration reached high levels, the Detoxifier population dominated the community (i.e., its relative fraction over 50%), suggesting that substrate toxicity considerably affected the structure of our consortium. Together, these experiments confirmed our simple rule proposed from mathematical modeling and suggested that the structure of a microbial community engaged in MDOL is governed by the concentration and toxicity of the substrate.

### 2.2 Substrate availability and toxicity shape the structure of the MDOL communities in spatially structured environments

In the above modeling and experiments, we investigated how substrate concentration and toxicity affect the structure of an MDOL community, principally by assuming that the substances and cells were well‐mixed in the system. However, microorganisms frequently grow in spatially structured environments^36^, ^38^. Previous studies reported that different physical characteristics between the well‐mixed and spatially structured systems significantly affected the structure of a community. These characteristics mainly include the differences in mass diffusion, as well as the spatial structure of the community^36^, ^39^, ^41^. Therefore, we set out to test whether our rule that the structure of a microbial community engaged in MDOL is governed by the concentration and toxicity of the substrate derived from a system that is well‐mixed can be expanded to estimate the structure of an MDOL community in spatially structured environments.

#### 2.2.1 Individual‐based (IB) modeling of the dynamics of an MDOL community

To simulate the dynamics of the MDOL community in a spatially structured environment, we built an IB model. The basic configuration of our IB model was identical to the framework of our ODE model. In addition, we assumed that the diffusion of *S*, *I*, and *P* was limited in the IB model, and mediated by their diffusion coefficients ($D_{s}$, $D_{i}$, and $D_{p}$). Details of the IB model are described in Supporting Information: S2.1–S2.4.

**Figure 5:**
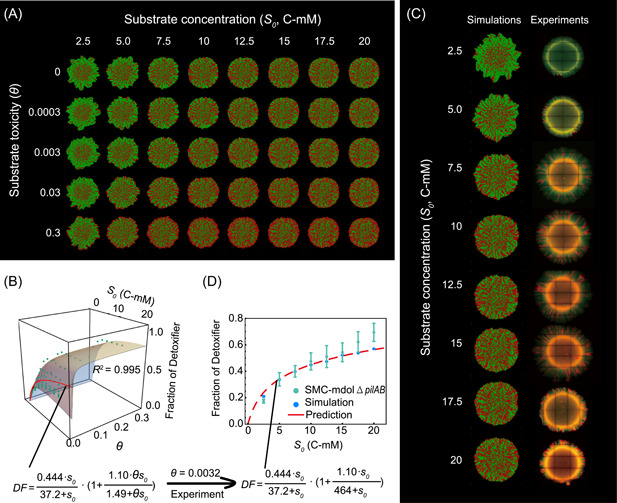
Substrate concentration and toxicity governing the structure of a microbial community engaged in MDOL in a spatially structured environment. (A) Representative colony patterns from Individual‐based (IB) modeling initialized with different substrate concentrations and toxicity. Detoxifier cells are shown in red, while Embezzler cells are shown in green. (B) Analysis of community composition in the expanding region of the colonies from IB simulations across eight different initial substrate concentrations and five different toxicity strengths. The plot shows how both substrate concentration and its toxicity collectively affect the steady‐state proportion of the Detoxifier. The green dots denote the simulated steady‐state fraction of the Detoxifier. The surface shows the plot of the best‐fitting function using Equation (2). The red line on the surface denotes the scenario *θ *= 0.0032, which is the degree of toxicity of salicylate obtained from experimental measurements. (C) Representative colony patterns from the pattern formation assays of SMC‐mdolΔ*pilAB*, as well as the IB simulations using the parameters matched with our synthetic system (Table S7), across eight different initial substrate concentrations. (D) The experimentally measured steady‐state fractions of Detoxifier in the expanding region of these colonies are consistent with those from mathematical predictions. Note that in the plots, substrate concentrations are shown in dimensional form (*S*_*0*_, C‐mM), but in the predictive functions, the fitting analyses were performed using its dimensionless form (*s*_*0*_).

To test our hypotheses, we ran the IB model using the parameters consistent with our experimental system (Table S7) but varied the degree of toxicity (*θ*) and initial concentration of the substrate (*s*_*0*_). We found that during colony growth, cell lineages of Detoxifier and Embezzler segregated at the frontiers, forming adjacent red and green cell sectors (Figure 5A,C; Supporting Information: Videos [Link], [Link], [Link], [Link]). Analysis of the spatial distribution of *S*, *I*, and *P* suggested that the development of this colony characteristic was mainly attributed to the “active layer effect” reported previously^42^. As *S* is generally supplied from the outside of the colony, a thin active cell layer was formed depending on the penetration of *S*, *I*, and *P* (Supporting Information: Videos [Link], [Link], [Link], [Link]). Consequently, community structures in the inoculated and expanding regions may differ. Accordingly, we separately analyzed the structures in the inoculated region and expanding region of the colonies (Figure S15). We found that with the growth of the colony, community structures in the inoculated region changed little, while the community structures in the expanding region shifted over time, gradually approaching a steady‐state (Figure S16). Therefore, we next investigated how substrate concentration and toxicity affect the steady‐state structures of the MDOL community in the expanding regions. The community structure in the expanding region was significantly affected by substrate concentration and toxicity, and was well estimated by the rule (Equation 2) we proposed for a well‐mixed system (Figures 5B and S17). This result indicated that the structure of the MDOL community in spatially structured environments can also be estimated by the proposed simple formula governed by substrate concentration and toxicity.

We also found that increasing substrate concentrations assisted the Detoxifier in obtaining more product from the environment, thus retaining higher intracellular product concentrations (Figure S18). Furthermore, Detoxifier cells possessed a lower intracellular concentration level of *S* than that of the Embezzler cells in our IB simulations (Figure S19). The higher rate of the first reaction, or lower *S* transport rate, also significantly increased the maximum benefit (${\mathrm{Ts}}_{\max}$) that Detoxifier cells can be obtained from substrate toxicity (Figure S20; correlation analysis *p* < 0.0001), which agreed with our results from ODE modeling. Therefore, the same mechanisms as found in the well‐mixed system are also applicable to explain why substrate concentration and toxicity affect the structure of the MDOL community in spatially structured environments.

#### 2.2.2 Experimental evaluation of our rule by culturing our synthetic microbial consortium in a spatially structured environment

We next experimentally tested our hypotheses in spatially structured environments. Several studies have reported that type IV pilus may affect microbial colony patterns^43^, ^45^. To directly focus on the effects of substrate concentration and toxicity and avoid the effects of pili, we deleted the *pilA* and *pilB* genes of both strains involved in our synthetic consortium. This design follows other studies that performed patterning experiments using nonmotile strains^46^, ^49^. The derived consortium was named SMC‐mdolΔ*pilAB*. This strain modification did not change the effects of substrate concentration and toxicity on the structures of the consortium in a well‐mixed system (Figure 4C; paired, two‐tailed, Student's *t*‐test: *p* = 0.3136), as well as the salicylate toxicity to the strains (Figure S13).

To test our hypotheses, we cultured SMC‐mdolΔ*pilAB* on an agarose surface to which salicylate was added at different concentrations. The experimentally observed colony patterns were very similar to those observed in the simulations (Figure 5C). We next separately assessed the structures of the consortium in both the inoculated region and expanding region of the colonies. We found that the proportion of Detoxifier population slightly shifted from 40.9 ± 3.5% to 60.0 ± 6.0% in the inoculated region (Figure S21), but it varied greatly from 17.4 ± 1.5% to 69.0 ± 7.0% in the expanding region (Figure 5D). The structure of the community in the inoculated region failed to be accurately captured by our mathematical model. We speculated that this inconsistency was due to the differences in the spatial dimension used in the model and tested in our experiments. During our IB simulations, cells only grew at the two‐dimensional level, so the cells located inside the inoculated region rarely grew, as a result of which the community structure within the region remained largely unchanged (keep at 1:1 ratio; Figure S16). In the experiments, the inoculated zone of the developed colony contains not only the inoculated cells but also the newly formed cells that grew in three‐dimensions. This difference limits the prediction power of our model for the inoculated region. Nevertheless, the experimental results of expanding region accurately fit our derived prediction function (Figure 5D), with a predicting power (adjusted *R* ^2^) of 0.982. This result suggests that the structure of the community containing only newly formed cells (the expanding region) can be accurately estimated by our proposed formula. Together, our simulations and experiments demonstrated that our rules on how substrate concentration and toxicity shape the structure of an MDOL community were applicable when this community grew in a spatially structured environment.

#### 2.2.3 The effects of substance diffusivity on the structure of the MDOL community

Although the structure of the MDOL community in spatially structured and well‐mixed environments can both be estimated by Equation (2), the estimated parameter values in the prediction functions derived from the ODE and IB models are slightly different (Figures 4 and 5), even if we applied identical parameters and equations in these two models (Supporting Information: S2.3). Through mathematical modeling, we found that limited mass diffusion is one of the major reasons that lead to this difference (see Supporting Information: S2.2 for detail). Our analyses suggest that a higher level of *P* diffusion favors Detoxifier (Figures S22 and S23), whereas increasing the diffusion level of *I* hindered Detoxifier (Figures S24 and S25).

**Figure 6:**
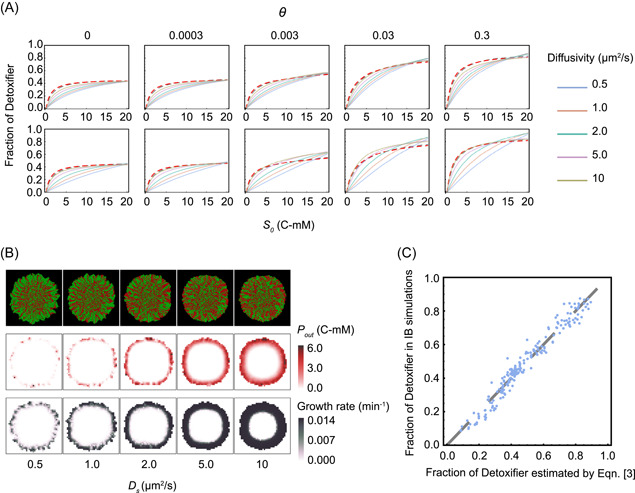
The effects of the diffusion level of substrate, intermediate, and product on the structure of the MDOL community. (A) The relationship between initial substrate concentration (*s*_*0*_) with the steady‐state proportion of Detoxifier cells in the expanding region of the colonies, across different substance diffusion levels (denoted by different curve colors) and different degrees of substrate toxicity (*θ*, denoted by five subgraphs). First row: diffusion levels of *S*, *I*, and *P* (i.e., *D*_i_, *D*_i_, and *D*_p_) were set to be identical and simultaneously modulated in the simulations. Second row: diffusion levels of *I* and *P* (*D*_i_ and *D*_p_) were set as default values shown in Table S7, while diffusion levels of S were modulated. Other parameters in these simulations were initialized with the default values shown in Table S7. The simulation data were then fit to Equation (2) to obtain the curves shown in the same plot. The adjust *R* ^2^ values for these fitting analyses range from 0.994 to 0.997. (B) Diffusion levels of substrate affected the thickness of the cell “active layer.” Representative colony images (first row), the corresponding distributions of the final product (second row), as well as the distributions of cell growth rates (third row) in the two‐dimensional plane at steady‐state, obtained from individual‐based simulations initialized with different diffusion levels of substrate. Shown are the results in which *s*_*0*_ was set to 10 C‐mM and *θ* was 0 (not including substrate toxicity). In the colony images, Detoxifier cells are shown in red, while Embezzler cells are shown in green. The thickness of the cell “active layer” is reflected by the thickness of the cell layer that possesses a positive growth rate (third row). (C) The linear correlation between the steady‐state frequencies of Detoxifier predicted by Equation (4) and the frequencies obtained by our Individual‐based simulations. The dashed line shows the linear curve in which the predicted results are completely identical to simulated results. The best‐fitting values of *ks*, *Fd*_*max*_, *kt*, *TS*_*max*_, *kd*_1_, and *kd*_2_, in this case, are 30.8, 0.446, 1.46, 1.05, 14,000, and 44.8, respectively.

In addition, we found that the diffusion level of the substrate has two opposing effects on the structure of the MDOL community. On the one hand, a higher diffusion level of *S* benefits the Detoxifier (Figure 6A, first row), by thickening the cell's “active layer”^49^ (Figure 6B), thus increasing the production and secretion of the final product by Embezzler cells. On the other hand, a higher diffusion level of *S* also decreases the fitness of the Detoxifier cells by modifying the concentration gradient of *S* around the two types of cells thus changing the relative toxic level of *S* (Figure 6A, second row; Figure S26). Combining these two effects, we introduced a new formula to estimate the structure of the MDOL community.

(3) $$\text{DF}=\frac{{\text{Fd}}_{\max}\text{∙}s_{0}}{\text{ks}\text{+}s_{0}}\text{∙}\left(1\text{+}\frac{{\text{Ts}}_{\max}\text{∙}\theta s_{0}}{\text{kt}\text{+}\theta s_{0}}\right)\text{∙}\left(\frac{s_{0}D_{s}}{{\text{kd}}_{1}\text{+}s_{0}D_{s}}\text{−}\frac{\theta D_{s}}{{\text{kd}}_{2}\text{+}\theta D_{s}}\right)$$

In this formula, $\frac{s_{0}D_{s}}{{\mathrm{kd}}_{1}+s_{0}D_{s}}$ represents an estimate of the positive effect of increasing substrate diffusion level via thickening cell “active layer,” related to the initial substrate concentration^49^ ($s_{0}$; Figure 6B); $\frac{\theta D_{s}}{{\mathrm{kd}}_{2}+\theta D_{s}}$ represents an estimate of the negative effect of increasing substrate diffusion level, influenced by the degree of toxicity of the substrate (Figure 6A; the second row). Equation (3) accurately estimated the structure of the MDOL community in our IB simulations (Figure 6C; *R* ^2 ^= 0.994). Overall, we concluded that substrate diffusivity is also fundamental to shaping the structure of the MDOL community in addition to the concentration and toxicity of the substrate.

## 3 DISCUSSION

Here, we show how substrate concentration and toxicity shape the structure of the microbial communities engaged in MDOL during the degradation of organic compounds. The population performing the first step is favored by both higher substrate concentration and its toxicity. This rule is applicable when the community grows both in a well‐mixed and spatially structured environment.

Recently, numerous studies have explored the strategy of dividing metabolic roles across different populations in a consortium toward the removal of organic pollutants^9^, ^12^, ^50^, ^53^. Our proposed rule may be expanded to forecast the structure of these consortia. For instance, one recent study reported that a bacterial consortium composed of *Leucobacter* sp. GP and *Achromobacter denitrificans* PR1 efficiently degrades an antibiotic, sulfamethoxazole in which the strain GP is responsible for the initial metabolism of the sulfamethoxazole (Detoxifier), and strain PR1 carries out the subsequent conversion (Embezzler)^16^. This study measured the structure of the community across a gradient of initial substrate concentrations and found that the proportion of the GP was positively correlated with the initial sulfamethoxazole concentration. This observation largely agrees with the idea derived from our model and experiments. The prediction of the structure of the community may greatly help to manage these communities for better performance^17^, ^30^, ^31^.

Our study also indicated that limited mass diffusion in spatially structured environments is another key factor in determining the structure of a community. This finding is reminiscent of recent studies proposing that limited mass diffusion plays a significant role in the structure of the communities engaged in other diffusion‐based interaction modes, including syntrophic exchange^39^, ^41^, ^54^, cross‐protection^55^, and “rock‐paper‐scissors” interaction^55^, ^56^. One important hypothesis from these studies is that limited mass diffusion is one possible way to privatize public benefit^39^, ^41^, ^57^. We found this hypothesis is also applicable in explaining the structuring of the community engaged in MDOL. On the one hand, limited mass diffusion helps the Embezzler population to privatize the final product for its own growth. On the other hand, it helps the Detoxifier population to privatize its benefit from detoxification. Therefore, limited mass diffusion may be a universally used avenue for microorganisms to maintain the private benefit of their activities in spatially structured environments.

In our IB modeling, we also found specific spatial patterns developed by the MDOL community. In agreement with previous studies^40^, ^58^, ^59^, when two populations engaged in MDOL, cells from the two populations are spatially more proximal to each other than in the scenario when the two populations did not exhibit defined interactions (Figure S27). In addition, we also found that the level of spatial proximity was governed by substrate concentration and toxicity (Figure S27). Interestingly, when the degree of substrate toxicity was high, the Detoxifier cells occupied the periphery of the growing colony, and formed a clear “ring” around the colony (Figures 5 and S28; Supporting Information: Video 3). The formation of this ring might be because the substrate was present at higher concentrations at the colony edge, and hence more toxic, thus largely favoring Detoxifier cells at the edge. These results suggest that substrate concentration and toxicity also govern the spatial distributions of different cells in the colony developed by the MDOL community, which may, in turn, affect the structure of such a community. Although we failed to observe this featured cell distribution in our experiments, one recent study found that an MDOL community that degrades toluene developed a similar “ring”‐shaped pattern as observed in our IB model^58^. Therefore, such cell distribution may represent a critical feature of the spatial patterns developed by an MDOL community that degrades toxic substrates.

While our study provides critical new insights into how the community engaged in MDOL assembles, a number of limitations need to be taken into consideration. First, our model analysis showed that substrate toxicity is vital in determining the structure of communities engaged in MDOL. However, due to the difficulties in manipulating the toxicity of the substrate (salicylate) *in vitro*, we were unable to experimentally compare the impact of the different degrees of toxicity on the structure of our community. Nevertheless, our model correctly predicts that simply increasing the initial substrate concentration is unlikely to shape a community dominated by the Detoxifier population, while the presence of substrate toxicity allows the “Detoxifier” population in the community to become dominant. Therefore, the observation that the Detoxifier population was able to dominate the synthetic consortium when supplied with a high concentration of salicylate, and the measured biotoxicity of salicylate strongly suggested that substrate toxicity should affect the structure of our synthetic microbial consortium. In agreement with this idea, our prediction functions involved in the degree of salicylate toxicity fit the experimental results very well. To further examine this idea, it is necessary to design a better system in which the toxicity of the substrate can be modulated.

Second, our ODE model suggests that apart from substrate concentration and toxicity, five other key parameters exist that exhibit considerable effects on the structure of an MDOL community. Here, we primarily focused on the effects of substrate, without analyzing in detail how all the seven key factors collectively determine the structure of the MDOL communities. Nonetheless, our analysis presented here suggests that biotic factors, such as the rate of the first reaction ($\alpha_{1}$), mass transport rate ($\gamma_{s}$, $\gamma_{i}$, $\gamma_{p}$), as well as the consumption rate of *P* (Cp), affected the structure of the community, namely by determining the value of parameters in Equation (2) (i.e., ${\mathrm{Fd}}_{\text{max}}$, ks, ${\mathrm{Ts}}_{\text{max}}$, and kt). For example, the value of Cp, $\gamma_{i}$, and $\gamma_{p}\;$regulates the community structure by affecting ${\mathrm{Fd}}_{\max}$ because ${\mathrm{Fd}}_{\max}$ strongly negatively correlates with Cp and positively correlates with $\gamma_{i}$ and $\gamma_{p}$ (Figure S8). The underlying mechanism may be that increasing the *P* consumption capacity (Cp) causes the Embezzler to release lower amounts of *P* to the environment, thus impairing the Detoxifier. Similarly, increasing the transport of the intermediate ($\gamma_{i}$) and product ($\gamma_{p}$) boosts the production and the leakiness of the end product, thus benefiting the Detoxifier. However, due to the difficulties in analytically solving nonlinear ODEs, in addition to the low efficiency of IB simulations^60^, a detailed quantitative understanding of how all these factors affect the structure of the MDOL community remains limited. Further studies may use more simplified models that combine these elements to provide a more general description of the principles governing the structuring of an MDOL community.

In addition, the MDOL system investigated in this study is built on the basic assumption that both degradation reactions were performed intracellularly. This assumption is true for many degradation pathways of organic compounds (such as bacterial degradation of PAHs^25^ and plastics^61^). Biochemical reactions that occur intracellularly help both the Detoxifier and Embezzler privatize their specific benefits, especially in well‐mixed environments. However, a number of degradation reactions are mediated by exoenzyme^62^, which means that these reactions occur in the extracellular space. If the first reaction in the MDOL is performed extracellularly, detoxification may become a cheatable public good^63^, ^64^.For example, the degradation of beta‐lactam occurs extracellularly, and thus may benefit the microbes that are not resistent to the related antibiotics^65^, ^66^. As a result, the degradation of the toxic substrate would immediately benefit both populations and does not give a specific benefit to the Detoxifier. Similarly, if the second reaction is performed extracellularly, the end product becomes a public good equally available to both strains. Therefore, we acknowledge that our framework may not be applicable to the scenario when any of the degradation reactions involved in the MDOL occur extracellularly. Nevertheless, limited mass diffusion can also act as an avenue for microorganisms to maintain their private benefit as we discussed before. From this perspective, benefits are allocated asymmetrically when the communities grow in spatially structured environments, even if the degradation reactions were catalyzed extracellularly^65^, ^67^. Thus, our results may also have implications for these scenarios.

Managing microbial communities engaged in MDOL recently emerges as an efficient and stable approach to removing the important pollutants that harm the natural environment, limit the agricultural output, and seriously threaten human health^9^, ^68^, ^70^. Our results demonstrate that the structure of a given community engaged in MDOL can be managed and quantitatively predicted from the abiotic factors present, for example, the concentration and toxicity of its substrate. These findings suggest that it is feasible to regulate microbial communities through the manipulation of specific environmental factors to address the grand challenges in environmental pollution and human health.

## 4 MATERIALS AND METHODS

### 4.1 Formulation and analyses of the ODE model

4.1.1

To simulate the dynamics of an MDOL community in a well‐mixed system, a mathematical model was formulated using ODEs. Here, the dimensionless forms of the models were presented. The detailed derivations of the models and choices of parameter values are described in Supporting Information: S1.

As described in Results section, a two‐step pathway was assumed to be implemented by MDOL between two populations (Figures 2A and 3A). For simplicity, the basic model was built based on five simple assumptions: (1) The systems are well mixed in each compartment (inside a cell or in the extracellular space); (2) transport of substrate (*S*), intermediate (*I*), and the final product (*P*) is mediated by passive diffusion; (3) *P* was assumed to be the sole and limited resource for the growth of the two populations and its consumption was calculated following Monod equations; (4) basic biological properties (the coefficients in Monod equations) regarding the growth of the two populations are identical since we only focused on the effects of abiotic factors; (5) when applicable, substrate toxicity was introduced by adding three different toxic terms to the growth equation (Table S3), dependent on intracellular *S* concentration of the corresponding population. The detailed justifications of our assumptions are listed in Supporting Information: S1.3. The dynamics of intracellular and extracellular *I* and *P* are given by

(4) $$\frac{{\text{ds}}_{1\text{,in}}}{\text{dτ}}\text{=}\;-\frac{\alpha_{1}}{1\text{+}s_{1\text{,in}}}s_{1\text{,in}}\text{+}{\text{γ}}_{s}\text{∙}\left(s_{\text{out}}\text{−}s_{1\text{,in}}\right)$$

(5) $$\frac{{\text{ds}}_{2\text{,in}}}{\text{dτ}}={\text{γ}}_{s}\text{∙}\left(s_{\text{out}}\text{−}s_{2\text{,in}}\right)$$

(6) $$\frac{{\text{di}}_{1\text{,in}}}{\text{dτ}}=\frac{\alpha_{1}}{1\text{+}s_{1\text{,in}}}s_{1\text{,in}}\text{−}{\text{γ}}_{\text{i}}\text{∙}\left({\text{i}}_{1\text{,in}}\text{−}{\text{i}}_{\text{out}}\right)$$

(7) $$\frac{{\text{di}}_{2\text{,in}}}{\text{dτ}}\text{=}\;-\frac{\alpha_{2}}{{\text{β}}_{2}\text{+}{\text{i}}_{2\text{,in}}}{\text{i}}_{2\text{,in}}\text{+}{\text{γ}}_{\text{i}}\text{∙}\left({\text{i}}_{\text{out}}\text{−}{\text{i}}_{2\text{,in}}\right)$$

(8) $$\frac{{\text{dp}}_{1\text{,in}}}{\text{dτ}}\text{=}\;-\frac{\text{Cp}}{{\text{β}}_{\text{g}}\text{+}{\text{p}}_{1\text{,in}}}{\text{p}}_{1\text{,in}}\text{+}{\text{γ}}_{\text{p}}\text{∙}\left({\text{p}}_{\text{out}}\text{−}{\text{p}}_{1\text{,in}}\right)$$

(9) $$\frac{{\text{dp}}_{2\text{,in}}}{\text{dτ}}=\frac{\alpha_{2}}{{\text{β}}_{2}\text{+}{\text{i}}_{2\text{,in}}}{\text{i}}_{2\text{,in}}\text{−}\frac{\text{Cp}}{{\text{β}}_{\text{g}}\text{+}{\text{p}}_{2\text{,in}}}{\text{p}}_{2\text{,in}}\text{+}{\text{γ}}_{\text{p}}\text{∙}\left({\text{p}}_{\text{out}}\text{−}{\text{p}}_{2\text{,in}}\right)$$

(10) $$\frac{{\text{ds}}_{\text{out}}}{\text{dτ}}\text{=}\;-{\text{x}}_{1}\text{∙}{\text{γ}}_{s}\text{∙}\left(s_{\text{out}}\text{−}s_{1\text{,in}}\right)\text{−}{\text{x}}_{2}\text{∙}{\text{γ}}_{s}\text{∙}\left(s_{\text{out}}\text{−}s_{2\text{,in}}\right)$$

(11) $$\frac{{\text{di}}_{\text{out}}}{\text{dτ}}={\text{x}}_{1}\text{∙}{\text{γ}}_{\text{i}}\text{∙}\left({\text{i}}_{\text{out}}\text{−}{\text{i}}_{1\text{,in}}\right)\text{−}{\text{x}}_{2}\text{∙}{\text{γ}}_{\text{i}}\text{∙}\left({\text{i}}_{\text{out}}\text{−}{\text{i}}_{2\text{,in}}\right)$$

(12) $$\frac{{\text{dp}}_{\text{out}}}{\text{dτ}}={\text{x}}_{2}\text{∙}{\text{γ}}_{\text{p}}\text{∙}\left({\text{p}}_{\text{out}}\text{−}{\text{p}}_{1\text{,in}}\right)\text{−}{\text{x}}_{1}\text{∙}{\text{γ}}_{\text{p}}\text{∙}\left({\text{p}}_{\text{out}}\text{−}{\text{p}}_{1\text{,in}}\right)$$

The growth of the two populations was modeled using a generalized logistic function with first‐order cell death:

(13) $$\frac{{\text{dx}}_{1}}{\text{dτ}}=\frac{\text{Cp}}{\text{bg}\text{+}{\text{p}}_{1\text{,in}}}{\text{p}}_{1\text{,in}}\text{y}{\text{t}}_{1}{\text{x}}_{1}\left(1\text{−}\frac{{\text{x}}_{1}\text{+}{\text{x}}_{2}}{\text{ρ}}\right)$$

(14) $$\frac{{\text{dx}}_{2}}{\text{dτ}}=\frac{\text{Cp}}{\text{bg}\text{+}{\text{p}}_{2\text{,in}}}{\text{p}}_{2\text{,in}}{\text{yt}}_{2}{\text{x}}_{2}\left(1\text{−}\frac{{\text{x}}_{1}\text{+}{\text{x}}_{2}}{\text{ρ}}\right)$$

The definitions and dimensionless methods of all variables are listed in Table S1. The definitions and dimensionless methods, as well as the value ranges of all the parameters involved in these equations, are listed in Table S2.

Details of the simulation and analysis protocols of our ODE model and the downstream analyses are described in Supporting Information: S1.4. Briefly, to solve the community dynamics of the MDOL community with given parameter sets, numerical simulations of our ODE model were performed using the *NDsolve* function of *Wolfram Mathematica*. The numerical solutions of all the variables, including the dynamics of mass (*S*, *I*, *P*) concentration and biomass, were recorded for further analyses. To perform simulations with numerous parameter sets, as well as the downstream analysis, custom *Mathematica* scripts were written mainly based on the *Do* loop function.

### 4.2 IB modeling

Our IB model was constructed based on the *gro* platform (https://github.com/liaupm/GRO‐LIA), a simulator designed by Gutiérrez and colleagues aiming to describe multicellular bacterial behavior^71^. The model aims to simulate the growth of a microbial colony composed of two populations who execute substrate degradation via MDOL on a surface. The model was formulated mainly using the same equations as our dimensional ODE model (Supporting Information: S1.1, Equations S1–S13) to characterize the intra‐ and extracellular dynamics of mass (*S*, *I*, *P*) concentration, as well as to calculate the rate of cell growth. Four main differences exist between our IB model and the ODE model: (1) the IB model was formulated on a spatially structured surface, and the diffusion of *S*, *I*, and *P* was limited; (2) mass dynamics was modeled at the single‐cell level; (3) the growth of both populations was modeled at the single‐cell level, and passive cell shoving during the cell growth was included; and (4) cells were inoculated in the center of the surface, and the entire community underwent “colony range expansion,” a process whereby the community immigrates outwards as a whole, driven by the force generated from cell growth and division (Figure S15). The mathematical framework formulating these four points is described in Supporting Information: S2.1. To implement our design of the IB model, custom codes were written in the *gro* language. Variables and Parameters in the IB model are summarized in Table S7. Details of the IB simulation workflow are described in Supporting Information: S2.

### 4.3 Genetic manipulation of the *P. stutzeri* strains

All *P. stutzeri* strains were engineered from a naphthalene‐degrading bacterial strain *P. stutzeri* AN10^72^. Genes that encode the key enzymes responsible for corresponding metabolic steps in the salicylate degradation pathway were knocked out to generate the *P. stutzeri* strains. The details of the genetic manipulation are described in Supporting Information: S3.

### 4.4 Liquid cultivation of our synthetic microbial communities

Liquid cultivation of our synthetic microbial communities was performed in 96‐well plates that contains 120 μl fresh minimum medium. Proportions of the two populations in the community were estimated by measuring the fluorescent intensity of the two strains involved using a microplate reader (Molecular Devices). Detailed protocols are described in Supporting Information: S4.

### 4.5 Colony pattern formation assays

Colony pattern formation assays were performed on the agarose surface in a Petri dish (60 mm in diameter). Images of the colony patterns were taken under a 5× objective using a Leica DM6000B fluorescence microscope (Leica Corporation) equipped with a LED fluorescence illuminator (Leica Corporation). The relative fraction of each population in the colonies was measured by image analysis, as well as the similar fluorescence‐measurement method as performed in liquid cultivation experiments. Detailed protocols are described in Supporting Information: S5.

### 4.6 Statistical analysis

Unless indicated otherwise, the number of replicates was three for each simulation, and six for each experiment. For comparative statistics, an unpaired, two‐tailed, Student's *t*‐test was performed in Wolfram Mathematica (version 12.4). To fit the data to the proposed function, the nonlinearmodelfit function of the Wolfram Mathematica (version 12.4) was applied.

## AUTHOR CONTRIBUTIONS

Miaoxiao Wang and Yong Nie were involved in the conceptualization of the study. Miaoxiao Wang constructed the mathematical models and performed computational simulations. Miaoxiao Wang designed the experiments. Miaoxiao Wang and Xiaoli Chen performed the experiments. Miaoxiao Wang analyzed the data and wrote the original draft. Yong Nie and Xiao‐Lei Wu edited the manuscript. Yong Nie, Yue‐Qin Tang, and Xiao‐Lei Wu raised the funding for the project.

## ETHICS STATEMENT

This article does not contain any studies with human participants or animals performed by any of the authors.

## CONFLICT OF INTERESTS

The authors declare no conflict of interests.

## Supporting information

Supporting information.

Supporting information.

Supporting information.

Supporting information.

Supporting information.

## Data Availability

All custom *Mathematica* codes used for ODE simulation and data analyses, as well as the source *gro* codes used for our IB simulations, are available at Github: https://github.com/RoyWang1991/MDOLcode/tree/master/MDOL-spatial.
